# Trends in Provision of Medications and Lifestyle Counseling in Ambulatory Settings by Gender and Race for Patients With Atherosclerotic Cardiovascular Disease, 2006-2016

**DOI:** 10.1001/jamanetworkopen.2022.51156

**Published:** 2023-01-19

**Authors:** Anthony Mufarreh, Amit J. Shah, Viola Vaccarino, Ambar Kulshreshtha

**Affiliations:** 1 Central Michigan University College of Medicine, Central Michigan University, Mt Pleasant; 2 Division of Cardiology, Department of Medicine, Emory University, Atlanta, Georgia; 3 Atlanta VA Health Care System, Decatur, Georgia; 4 Department of Epidemiology, Rollins School of Public Health, Emory University, Atlanta, Georgia; 5 Division of Family and Preventative Medicine, Emory University School of Medicine, Atlanta, Georgia

## Abstract

**Importance:**

Atherosclerotic cardiovascular disease (ASCVD) continues to be highly prevalent in the US. The 2013 American College of Cardiology and American Heart Association (ACC/AHA) treatment guidelines reevaluated evidence-based practices for reduction of ASCVD in men and women from high-quality randomized trials and meta-analyses recommending the use of statin therapy, aspirin prescription, and lifestyle counseling for adults with ASCVD. Population trends in secondary prevention strategies for patients with ASCVD among primary care settings is currently lacking, limiting ability to evaluate impact of guideline implementation.

**Objective:**

To examine temporal and sociodemographic trends in secondary prevention strategies in patients with ASCVD between 2006 and 2016 in a nationally representative, ambulatory care database.

**Design, Setting, and Participants:**

This cross-sectional study analyzed data from the National Ambulatory Medical Care Survey (NAMCS), which is an annual survey conducted to represent the national US population and contains information on ambulatory office-based patient visits, including medical conditions, services provided, and demographic characteristics. Participants were adults aged 21 years and older with prevalent ASCVD identified via International Classification of Disease codes between 2006 and 2016. Data were extracted and analyzed in March 2021.

**Main Outcomes and Measures:**

Data were separated by calendar year pre-2013 (2006 to 2013) and post-2013 (2014 to 2016). Outcomes included statin therapy, aspirin prescription, and lifestyle counseling (eg, nutrition, exercise, weight reduction) service provided at clinic visits.

**Results:**

There were 11 033 visits for adults with ASCVD, representing a weighted total of 275.3 million visits nationwide; 40.7% (112.1 million [weighted]) were women, 9.2% (25.4 million [weighted]) were Hispanic, 9.9% (19.0 million [weighted]) were non-Hispanic Black, 90.1% (172.7 million [weighted]) were non-Hispanic White, and 40.6% (112.1 million [weighted]) were from cardiology clinics. Of 11 033 patient visits, 5507 patients (49.9%) were prescribed statin therapy, 5165 patients (46.8%) were using aspirin, 2233 patients (20.2%) received lifestyle counseling. Statin therapy increased from 9.3 million individuals (45.3%) in 2006 to 14.9 million individuals (46.5%) in 2016, and aspirin prescriptions increased from 8.5 million individuals (41.3%) in 2006 to 15.2 individuals (47.5%) in 2016. Women were less likely than men to receive medications for secondary prevention: among women, 48.8 million (43.3%) received statins (vs 85.9 million men [52.7%]), 44.7 million (39.8%) received aspirin (vs 79.1 million men [48.5%]), and 25.7 million (22.9%) received lifestyle counseling services (vs 37.5 million men [23.0%]).

**Conclusions and Relevance:**

These findings suggest only modest increases in statin and aspirin prescription since 2006; however, lifestyle counseling use decreased in recent years. Women and Black patients continued to be less likely to receive secondary prevention ASCVD treatment. Adherence to guideline-directed secondary prevention recommendations remained low (less than 50%) in patients with ASCVD, especially with regards to lifestyle counseling, suggesting the need for more implementation research.

## Key Points

**Question:**

What are the trends and sociodemographic variations in the use of secondary prevention treatments for US adults with atherosclerotic cardiovascular disease (ASCVD) in ambulatory settings prior to and following the 2013 American College of Cardiology and American Heart Association guidelines?

**Findings:**

In this cross-sectional study using nationally representative sample of 11 033 visits for adults with ASCVD, representing a weighted total of 275.3 million visits, statin therapy, aspirin prescription, and lifestyle counseling rates remained low. There was an increase in statin and aspirin prescriptions following the 2013 American College of Cardiology and American Heart Association guidelines, and women and Black patients continued to have lower rates of ASCVD prevention treatments.

**Meaning:**

These findings suggest low use of secondary cardiovascular prevention strategies among patients with ASCVD and persisting gender and racial disparities.

## Teaser

This cross-sectional study investigates temporal and sociodemographic trends in secondary prevention strategies in patients with atherosclerotic cardiovascular disease (ASCVD) between 2006 and 2016 in a nationally representative ambulatory care database.

## Introduction

Atherosclerotic cardiovascular disease (ASCVD) consists of coronary artery disease, ischemic stroke, transient ischemic attack, and peripheral artery disease and is a leading cause of morbidity and mortality in the United States.^1^ More than 300 000 deaths annually in the US are attributed to ASCVD.^2^ Additionally, patients with ASCVD face exceedingly high health care utilization costs from suboptimal treatment, along with a high burden of disease.^3^

In 2013, the American College of Cardiology and American Heart Association (ACC/AHA) Guideline on the Treatment of Blood Cholesterol to Reduce Atherosclerotic Cardiovascular Risk in Adults was published and serves as the updated treatment guidelines for secondary prevention of ASCVD. Guidelines expanded risk groups that were eligible and would benefit from statin therapy and lifestyle counseling, such as increased physical activity and healthy nutrition, to all adults with known ASCVD, regardless of low-density lipoprotein cholesterol levels.^4,5,6^ This was projected to increase adults eligible for statin by 12.8 million.^7^ Low-dose aspirin use has also been previously recommended for secondary prevention in patients with ASCVD.^8,9^

Previous studies that have used medical expenditure data to monitor trends in statin therapy, exercise, and lifestyle counseling trends have documented either modest or negligible improvements in treatment among high-risk groups.^10,11^ Using data from an ambulatory care survey, aspirin has been previously shown to be underused across all cardiovascular risk groups.^12^ This prior work was limited to medical expenditure data, with limited research documenting population trends of secondary prevention strategies in ambulatory care in recent years. Primary care practitioners are well-positioned to reduce cardiovascular risk factors due to continuity with patients and comprehensiveness of care. They also see a higher volume of patients with chronic diseases and people from minority racial and ethnic groups, including Black and Hispanic patients, positioning them optimally to improve health disparities among these populations.^13^

The current study uses a large, national, cross-sectional survey of ambulatory care visits of patients with ASCVD from 2006 to 2016 to examine temporal trends of secondary prevention strategies: statin therapy, aspirin prescription, and lifestyle counseling. We aimed to describe changes in secondary prevention following the ACC/AHA published guidelines in 2013 and assessed differences across sociodemographic groups.

## Methods

This cross-sectional study uses data from the National Ambulatory Medical Care Survey (NAMCS), which has been approved by the National Center for Health Statistics (NCHS) Research Ethics Review Board. Waivers of the requirements to obtain informed consent of patients and authorization for release of patient medical record data were granted due to lack of personal identifiable information collected. Data are presented descriptively following the Strengthening the Reporting of Observational Studies in Epidemiology (STROBE) reporting guideline.^14^

### Population

The NAMCS was formed in 1973 to create national estimates of ambulatory medical care services provided in the United States.^15^ Conducted by the NCHS, NAMCS provides nationally representative information on ambulatory office-based visits to non–federally employed physicians engaged in direct patient care. Nonpatient care specialties (eg, anesthesiology, pathology, and radiology) are excluded. Offices are sampled by specialty and geography, then randomly assigned a 1-week reporting period, recording services offered. Each physician was randomly assigned 1 reporting week to collect and report office visits. From this list, electronic patient records were again randomly sampled to ensure a systematic random sample was taken from each physician office. Demographic, comorbidity, current medications, and lifestyle counseling services were recorded for each visit, as well as sample weights to provide nationally representative estimates. Patient race and ethnicity were collected as patient self-report in medical records and included to describe the sample. Race and ethnicity categories included Hispanic, non-Hispanic Black, and non-Hispanic White. Race and ethnicity were assessed to examine trends by different demographic groups. The national survey is conducted and administered annually by the NCHS in the Centers for Disease Control and Prevention. Previous iterations of NAMCS have been validated against other data sources.^16^

Inclusion criteria for study participants included adults (aged ≥21 years) with a history of ASCVD who attended an ambulatory care visit between 2006 and 2016. ASCVD was defined as myocardial infarction, angina, coronary revascularization, ischemic stroke, transient ischemic attack, or peripheral artery disease, identified from the *International Classification of Diseases, Ninth Revision, Clinical Modification* (*ICD-9-CM*) from 2006 to 2015, and comparable *International Statistical Classification of Diseases, Tenth Revision, Clinical Modification (ICD-10-CM)* for 2016 from diagnosis list of sampled visits (eTable 1 in the Supplement).

### Measures

Statin therapy (ie, atorvastatin, fluvastatin, lovastatin, pitavastatin, pravastatin, and rosuvastatin) and aspirin prescription were identified using reclassified patient records using NAMCS Lexicon Plus generic component classifications database, a proprietary database of Cerner Multum used by the National Health and Nutrition Examination Survey.^17^ Lifestyle counseling refers to information given to patients during visit or referral to other health professional for purpose of behavioral change and includes weight reduction, nutrition counseling, and exercise counseling. NAMCS data processes missing data using single, sequential regression imputation methodology for any missing race and ethnicity data, using physician specialty, geographic region, and 3-digit *ICD-10-CM* code for primary diagnosis.

Patient visit characteristics were assessed using sample weight by adults with ASCVD. Trends in the overall population by each of the secondary prevention strategies were assessed using sample weights. Univariate analysis for each strategy was then stratified by sociodemographic factors and comorbidities of interest as the independent variables.

### Statistical Analysis

For all 3 secondary prevention strategies, temporal trends by year were performed from 2006 to 2016. For an assessment of the uptake of the 2013 ACC/AHA guidelines, we combined study years 2006 to 2013 to represent the period prior to the guideline change (referred henceforth as pre-2013) and 2014 to 2016 as post implementation (post-2013). Each study year was an individual sample, and they were not linked across. Data from each year were pooled to form the dependent variable post-2013, representing pooled study cycles 2006 to 2013 and 2013 to 2016. Sample weights were applied to all observations and summed across study years to produce frequencies. Subgroup analysis was performed by sociodemographic (gender, race and ethnicity, age), physician specialty, census region, insurance, and comorbidities. Adjusted odds ratios (aORs) along with corresponding 95% Wald-CIs are reported for logistic regression analysis involving secondary prevention outcomes including statin therapy, aspirin prescription, and lifestyle counseling, respectively, comparing odds of receiving the specific secondary prevention intervention post-2013 compared with pre-2013. All models adjusted for gender, race and ethnicity, age, specialty, geographic region, insurance type, obesity, hypertension, diabetes, cerebrovascular disease, and chronic kidney failure. Each observation represents a specific ambulatory care visit, all of which contain individual sample weight. Estimates were created by aggregation of sample weight variable in pooled sample (pre-2013 vs post-2013). The continuity of NAMCS sample weight variable across study years enabled creation of pooled statistics. Statistical analyses accounted for sample weights and complex sample design of NAMCS. Differences were evaluated using 2-sided significance tests set at a *P* < .05. Statistical analysis performed in March 2021 using R version 3.6.2 (R Project for Statistical Computing) *survey* package.^18^

## Results

From 2006 to 2016, 11 033 visits involved adults with ASCVD, representing 275.3 million visits (weighted). Overall cohort median (IQR) age was 70 (53-87) years; 40.7% of visits (112.1 million [weighted]) were among women; 9.2% of visits (25.4 million [weighted]) were among Hispanic patients, 9.9% of visits (19.1 million [weighted]) were among non-Hispanic Black patients, and 90.1% of visits (172.7 million [weighted]) were among non-Hispanic White patients; and 40.6% of visits (111.6 million [weighted]) were from cardiology clinics (Table 1). Patients with ASCVD had a high prevalence of comorbidities including 191.2 million patients (69.5%) with hypertension, 158.2 million patients (57.5%) with hyperlipidemia, 79.8 million patients (29.0%) with diabetes, and 29.4 million patients (10.7%) with obesity. Of patients with ASCVD, 5507 (49.0%) were statin users, 5165 (46.8%) were using aspirin, and 2233 (20.2%) received lifestyle counseling.

**Table 1.** Secondary Prevention Interventions Among Patients With Atherosclerotic Cardiovascular Disease, Aged at Least 21 Years by 2013 American College of Cardiology and American Heart Association Guidelines^a^

Variable	No. (weighted), millions (%)					
	Pre-2013 (2006-2013) (n = 181.3)			Post-2013 (2014-2016) (n = 94.0)		
	Statin	Aspirin	Lifestyle counseling	Statin	Aspirin	Lifestyle counseling
Total (weighted)	83.6 (46.1)	76.7 (42.3)	42.7 (23.5)	50.9 (54.1)	47.1 (50.1)	20.5 (21.9)
Gender						
Men	53.6 (50)	48.9 (45.7)	26.3 (24.6)	32.5 (57.7)	30.2 (53.8)	11.2 (20)
Women	30.2 (40.6)	27.8 (37.4)	16.4 (22)	18.4. (48.8)	16.9 (44.7)	9.3 (24.7)
Race and ethnicity						
Hispanic	6.9 (44.4)	6.1 (39.4)	4.0 (25.8)	4.3 (43.3)	4.1 (41.6)	2.8 (28.3)
Non-Hispanic Black	4.8 (41.6)	4.0 (35.3)	2.5 (22.1)	3.2 (41.8)	3.8 (49.7)	2.1 (27.4)
Non-Hispanic White	53.5 (46.9)	49.3 (43.2)	28.2 (24.7)	32.5 (55.5)	30.0 (51.2)	11.9 (20.3)
Age, y						
21-39	0.6 (23.8)	0.6 (24.7)	0.5 (21.1)	0.2 (7.5)	0.6 (29)	0.7 (33.1)
40-49	3.2 (33)	3.8 (39.3)	2.2 (22.6)	1.5 (44.5)	1.4 (40)	0.7 (20.3)
50-59	11.1 (43.7)	11.2 (43.9)	7.0 (27.6)	6.5 (55.4)	6.3 (54.3)	3.6 (31)
60-75	36.5 (49.3)	33.3 (44.9)	18.0 (24.3)	23.2 (54.9)	22.0 (52.1)	9.0 (21.2)
≥75	32.2 (46.3)	27.8 (40)	14.9 (21.5)	19.5 (56.6)	16.8 (48.6)	6.5 (19)
Specialty						
Family	12.6 (40.1)	8.4 (26.9)	6.5 (20.6)	10.8 (57.2)	8.3 (43.9)	5.8 (30.8)
Internal	18.8 (47.8)	15.4 (39.1)	11.6 (29.4)	10.0 (61.1)	8.6 (52.7)	4.5 (27.4)
Cardiology	41.3 (54.8)	41.3 (54.9)	20.0 (26.6)	27.5 (51.8)	28.0 (52.6)	9.5 (17.9)
Census region						
Northeast	17.4 (45.8)	16.3 (42.9)	10.5 (27.5)	4.5 (60.6)	3.7 (49.9)	0.9 (12.2)
Midwest	19.3 (50.2)	17.5 (45.5)	8.0 (20.8)	4.4 (61.5)	4.5 (63.2)	1.6 (23.2)
South	32.3 (43.5)	30.7 (41.3)	17.2 (23.2)	6.8 (57)	5.4 (45.1)	1.8 (15.2)
West	14.6 (47.8)	12.2 (39.8)	7.0 (22.9)	3.2 (66.3)	2.5 (52.4)	0.8 (17.4)
Insurance						
Private	25.3 (46.9)	25.1 (46.6)	13.9 (25.7)	12.2 (49.3)	13.2 (53.3)	5.3 (21.4)
Medicare	50.9 (47)	44.5 (41.1)	24.6 (22.7)	32.1 (56.3)	27.6 (48.5)	12.0 (21)
Medicaid or CHIP	2.7 (35)	2.4 (30.9)	1.9 (25.1)	2.0 (38.7)	2.3 (45.7)	1.7 (32.8)
Obesity^b^	9.1 (49.5)	8.2 (44.7)	8.9 (48.6)	6.9 (62.3)	6.2 (55.8)	4.3 (38.9)
Hypertension	59.0 (48.4)	53.7 (44.1)	32.1 (26.3)	40.9 (59.1)	37.2 (53.7)	14.9 (21.5)
Diabetes	23.1 (47.6)	18.4 (38)	14.2 (29.3)	18.3 (58.2)	16.4 (52.4)	7.7 (24.6)
Hyperlipidemia	56.6 (57.3)	48.7 (49.3)	29.0 (29.4)	38.3 (64.2)	33.3 (55.9)	13.8 (23.2)
Cerebrovascular disease	14.9 (36.4)	14.2 (34.8)	7.2 (17.6)	9.9 (50.7)	9.2 (46.9)	3.2 (16.4)
Tobacco use^c^	9.8 (44.3)	9.2 (41.5)	5.0 (22.6)	6.4 (53.9)	6.0 (50.4)	2.4 (20.3)
Kidney failure	3.8 (43.6)	3.4 (39.2)	2.5 (28.9)	6.3 (66.8)	4.3 (44.9)	2.5 (26.5)

Abbreviation: CHIP, Children’s Health Insurance Program.

^a^ Demographic information by pre-2013 (2006-2013) and post-2013 (2014-2016) for each secondary prevention intervention.

^b^ Body mass index, calculated as weight in kilograms divided by height in meters squared, greater than or equal to 30.

^c^ Smoking cigarettes or cigars, using snuff, or chewing tobacco.

Statin prescriptions increased marginally from 45.3% in 2006 to 46.5% in 2016, reaching its highest in 2014 at 60.3% soon after the new 2013 guidelines were released, but then declined. Aspirin prescription increased from 41.3% in 2006 to 47.5% in 2016. However, lifestyle counseling use decreased from 33.0% in 2006 to 22.3% in 2016, reaching its lowest in 2012 at 13.8% (Figure; eTable 2 in the Supplement).

**Figure.**
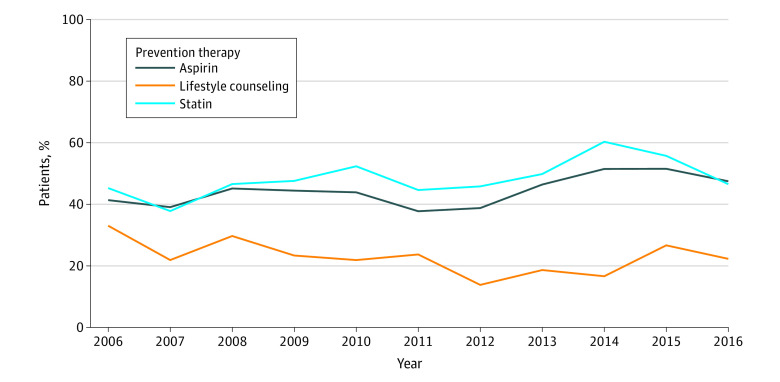
Trends in Secondary Prevention Therapies Among Patients With Atherosclerotic Cardiovascular Disease in Ambulatory Settings, 2006 to 2016 Temporal trends from 2006 to 2016 of each secondary prevention intervention among adult patients with atherosclerotic cardiovascular disease in the National Ambulatory Medical Care Survey.

Overall, women were less likely than men to receive secondary prevention lifestyle counseling (statins: 48.6 million women [43.3%] vs 85.9 million men [52.7%]; aspirin: 44.6 million women [39.8%] vs 79.1 million men [48.5%]; lifestyle counseling: 25.7 million women [22.9%] vs 37.5 million men [23.0%]) (Table 1). Non-Hispanic Black patients received less lifestyle counseling than non-Hispanic White patients. Among non-Hispanic Black patients, 7.9 million (41.6%) received statins, 7.8 million (41.1%) received aspirin, and 4.6 million (24.2%) received lifestyle counseling, compared with 58.9 million White patients (49.8%) receiving statins, 79.3 million White patients (45.9%) receiving aspirin, and 40.1 million White patients (23.3%) receiving lifestyle counseling. Patients seen at family medicine practices were less likely to receive statins (23.3 million patients [46.5%]) or aspirin (16.7 million patients [33.3%]) compared with internal medicine (statins: 28.8 million patients [51.7%]; aspirin: 24.0 million patients [43.1%]) and cardiology (statins: 62.1 million patients [55.6%]; aspirin: 62.6 million patients [56.2%]) practices, with lifestyle counseling highest among patients treated at internal medicine clinics (12.3 million patients [24.4%]) and lowest among those treated at cardiology clinics (27.0 million patients [24.2%]) (Table 1).

Statin use increased from 83.6 million patients (46.1%) to 50.9 million patients (54.1%) (Table 1). The gender gap in statin use was present and persistent but did decrease, with men at 53.6 million (50%) and women at 30.2 million (40.6%) pre-2013, compared with men at 32.5 million (57.7%) and women at 18.4 million (48.8%) post-2013.

Aspirin prescription increased by 7.8 percentage points from 76.6 million patients (42.3%) in pre-2013 to 47.1 million patients (50.1%) post-2013 (Table 1). Rates of aspirin prescription differed by gender by 8.3 percentage points (48.9 million men [45.7%] vs 27.8 million women [37.4%]) in pre-2013 to 9.1 percentage points (30.2 million men [53.8%] vs 16.9 million women [44.7%]) in post-2013. Adults aged 50 to 59 years with ASCVD experienced the greatest increase in aspirin prescription post-2013, with an increase of 10.4 percentage points (11.2 million patients [43.9%] pre-2013 vs 6.3 million patients [54.3%] post-2013).

Lifestyle counseling use decreased by 1.6 percentage points from 42.7 million patients (23.5%) pre-2013 to 20.5 million patients (21.9%) post-2013. Adults aged 21 to 39 years experienced the largest increase in lifestyle counseling use, by 12 percentage points from 0.5 million patients (21.1%) pre-2013 to 0.7 million patients (33.1%) post-2013. Most age groups had decreases in lifestyle counseling use, except patients aged 50 to 59 years (Table 1).

Family and internal medicine specialty visits had increased statin therapy pre-2013 compared with post-2013, with family clinic visits increasing by 17.2 percentage points, from 12.6 million patients (40.1%) pre-2013 to 10.8 million patients (57.2%) post-2013, and internal medicine visits increasing by 13.3 percentage points, from 18.8 million patients (47.8%) pre-2013 to 10.0 million patients (61.1%) post-2013. For aspirin prescription, family medicine clinics increased prescriptions by 17.0 percentage points, from 8.4 million patients (26.9%) pre-2013 to 8.3 million patients (43.9%) post-2013; internal medicine clinics increased prescriptions 13.6 percentage points, from 15.4 million patients (39.1%) pre-2013 to 8.6 million patients (52.7%) post-2013; while lifestyle counseling only increased in family medicine visits, with a 10.2–percentage points change from 6.5 million patients (20.6%) pre-2013 to 5.8 million patients (30.8%) post-2013. Cardiology practice clinics decreased prescriptions in all 3 secondary prevention strategies, with statin therapy decreasing by 3 percentage points, from 41.3 million patients (54.8%) pre-2013 to 28.0 million patients (52.6%) post-2013; aspirin prescriptions decreasing by 2.3 percentage points, from 41.3 million patients (54.9%) pre-2013 to 28.0 million patients (52.6%) post-2013; and lifestyle counseling decreasing by 8.7 percentage points, from 20.0 million patients (26.6%) pre-2013 to 9.5 million patients (17.9%) post-2013 (Table 1).

In regression analyses, the odds of secondary prevention post-2013 were higher for statin therapy (aOR, 1.29; 95% CI, 0.99-1.66) and aspirin prescription (aOR, 1.44; 95% CI, 1.11-1.87) but decreased for lifestyle counseling (aOR, 0.81; 95% CI, 0.58-1.12). Women were less likely than men to receive statin therapy (aOR, 0.79; 95% CI, 0.68-0.92) and aspirin prescription (aOR, 0.81; 95% CI, 0.7-0.95). Non-Hispanic Black patients were less likely to receive statin therapy compared with non-Hispanic White patients (OR, 0.72; 95% CI, 0.57-0.92); however, this was attenuated in adjusted models. Across insurance type recipients, Medicaid and Children’s Health Insurance Program recipients were the least likely to receive statin therapy (aOR, 0.63; 95% CI, 0.47-0.84) or aspirin (aOR, 0.61; 95% CI, 0.41-0.93) compared with private insurance recipients, but again this difference attenuated on adjusted models. No statistically significant changes post-2013 for lifestyle counseling were found by gender, race and ethnicity, or insurance type (Table 2).

**Table 2.** Multivariate Regression of Secondary Prevention Interventions Among Patients With Atherosclerotic Cardiovascular Disease, Aged at Least 21 Years by 2013 ACC/AHA Guidelines^a^

Variable	OR (95% CI)					
	Statin		Aspirin		Lifestyle counseling	
	Unadjusted	Adjusted	Unadjusted	Adjusted	Unadjusted	Adjusted
2013 ACC/AHA						
Pre-2013	1 [Reference]	1 [Reference]	1 [Reference]	1 [Reference]	1 [Reference]	1 [Reference]
Post-2013	1.38 (1.07-1.77)	1.48 (1.15-1.91)	1.37 (1.07-1.75)	1.37 (1.06-1.76)	0.91 (0.68-1.22)	0.66 (0.46-0.95)
Gender						
Men	1 [Reference]	1 [Reference]	1 [Reference]	1 [Reference]	1 [Reference]	1 [Reference]
Women	0.69 (0.61-0.77)	0.77 (0.67-0.9)	0.7 (0.62-0.8)	0.76 (0.65-0.89)	0.99 (0.85-1.16)	0.92 (0.79-1.07)
Race and ethnicity						
Non-Hispanic Black	0.72 (0.57-0.92)	0.9 (0.68-1.19)	0.82 (0.6-1.11)	0.8 (0.58-1.1)	1.05 (0.76-1.47)	0.95 (0.7-1.3)
Non-Hispanic White	1 [Reference]	1 [Reference]	1 [Reference]	1 [Reference]	1 [Reference]	1 [Reference]
Age, y						
21-39	1 [Reference]	1 [Reference]	1 [Reference]	1 [Reference]	1 [Reference]	1 [Reference]
40-49	2.87 (1.65-5.01)	1.38 (0.69-2.79)	1.79 (0.96-3.37)	1.84 (0.97-3.46)	0.78 (0.36-1.7)	0.82 (0.35-1.93)
50-59	4.59 (2.64-7.97)	1.89 (1.0-3.6)	2.45 (1.36-4.41)	2.24 (1.28-3.89)	1.11 (0.55-2.24)	0.99 (0.46-2.14)
60-75	5.37 (3.19-9.04)	2.52 (1.33-4.79)	2.49 (1.36-4.57)	2.06 (1.21-3.53)	0.84 (0.4-1.74)	0.76 (0.34-1.69)
≥75	5.03 (2.99-8.5)	2.33 (1.21-4.49)	2.06 (1.13-3.76)	1.99 (1.14-3.46)	0.72 (0.35-1.5)	0.65 (0.29-1.45)
Specialty						
Family	1 [Reference]	1 [Reference]	1 [Reference]	1 [Reference]	1 [Reference]	1 [Reference]
Internal	1.23 (0.95-1.6)	1.29 (0.97-1.71)	1.52 (1.15-2.0)	1.48 (1.07-2.04)	1.25 (0.86-1.82)	1.57 (1.08-2.28)
Cardiology	1.33 (1.04-1.7)	1.77 (1.45-2.17)	2.34 (1.82-3.01)	3.17 (2.44-4.13)	0.92 (0.66-1.29)	1.27 (0.88-1.83)
Geographic region						
Northeast	1 [Reference]	1 [Reference]	1 [Reference]	1 [Reference]	1 [Reference]	1 [Reference]
Midwest	1.16 (0.94-1.44)	1.34 (1.06-1.7)	1.19 (0.94-1.51)	1.38 (1.07-1.77)	0.81 (0.56-1.15)	0.7 (0.47-1.05)
South	0.89 (0.74-1.08)	0.98 (0.78-1.21)	0.92 (0.73-1.14)	1.01 (0.78-1.3)	0.85 (0.57-1.26)	0.74 (0.48-1.16)
West	1.09 (0.88-1.35)	1.14 (0.86-1.52)	0.91 (0.67-1.22)	0.93 (0.63-1.36)	0.85 (0.57-1.28)	0.94 (0.58-1.54)
Insurance type						
Private	1 [Reference]	1 [Reference]	1 [Reference]	1 [Reference]	1 [Reference]	1 [Reference]
Medicare	1.1 (0.96-1.18)	0.97 (0.81-1.17)	0.82 (0.71-0.94)	0.89 (0.73-1.09)	0.88 (0.74-1.06)	0.94 (0.76-1.15)
Medicaid/CHIP	0.63 (0.47-0.84)	0.95 (0.66-1.37)	0.61 (0.41-0.93)	0.76 (0.48-1.2)	1.22 (0.74-1.99)	1.18 (0.75-1.87)
Other	0.77 (0.52-1.14)	1.12 (0.7-1.79)	0.63 (0.43-0.91)	0.68 (0.39-1.18)	0.73 (0.43-1.26)	0.51 (0.25-1.02)

Abbreviations: ACC/AHA, American College of Cardiology and American Heart Associations; CHIP, Children’s Health Insurance Program.

^a^ Multivariable logistic regression comparing odds of receiving each secondary prevention intervention in pre-2013 to post-2013. Adjust models controlled for gender, race and ethnicity, age, specialty, geographic region, and insurance type.

## Discussion

In a this cross-sectional study using a nationally representative survey of office-based patient visits in adults with ASCVD from 2006 to 2016, secondary prevention strategies showed significant underuse. Statin therapy, aspirin prescription, and lifestyle counseling remained low in high-risk groups, with modest increases in statin therapy and aspirin prescription since 2006. Although there were improvements in gender and racial and ethnic differences in statin therapy and aspirin prescription observed post-2013, secondary prevention remained lower in women and non-Hispanic Black patients.

Statin therapy, aspirin prescription, and lifestyle counseling have been previously reported as underused in patients with ASCVD. Prior studies have found lower use of statin therapy among high-risk patients in both privately insured and Medicare enrollees, with an estimated rate of 59.9%.^19^ Data from the Medical Expenditure Panel Survey from 2 studies covering 2002 to 2013 and 2008 to 2016 showed little to no changes in statin therapy prevalence over time, aligned with the current study findings.^10,20^ Gender and racial gaps in statin therapy use have also been described, with female and Black patients having lower odds of receiving any statin therapy.^19^ Our study highlights the persistence of both gender and racial gaps in statin therapy use among patients with ASCVD among primary care settings, which may be a result of frequent changes in national guidelines and fewer programs that focus on implementation.

Aspirin prescription has long been established for both primary and secondary prevention of cardiovascular disease, however, use remains low.^8,21^ Primary care offers a key opportunity to expand aspirin prescription in patients with high-risk ASCVD.

Lifestyle counseling is a long-standing cardio-protective lipid-lowering treatment, however use remains very low.^8^ A previous study among peripheral artery disease patients in NAMCS and NHAMCS found only 22% of visits involved exercise or diet lifestyle counseling.^22^ Decreasing time slots for patient encounters, as well as lack of adequate clinical training in nutrition and physical activity counselling and behavior change has been identified as barriers in primary care setting and may explain low use.^23^ More programs that focus on physician education in applying guidelines to patients, electronic medical record reminders, and assessing patient resistance routinely at office visits could help mitigate some of the barriers in increasing use of secondary ASCVD preventive therapy.^24^

### Limitations

There are several limitations to this study. Data may be incomplete, since lifestyle counseling may have been underreported or misclassified. Study year was limited up to 2016 and was not able to account for updated guidelines. This study is cross-sectional and was therefore not able to uncover the causative reasons for existing disparities between groups. There was no information regarding statin or aspirin intolerance and other reasons why physicians or patients may have opted not to be on these treatments. The data are based on self-report by practices, and we also lack data on adherence. Several relevant confounders in regression analysis were unavailable in NAMCS, including statin dose, intensity, treatment adherence, education level, location of practice, participant income, and sufficient sample of racial and ethnicity groups beyond those presented.

## Conclusions

The present study suggests suboptimal use of statin therapy, aspirin prescription, and lifestyle counseling in ambulatory settings among patients with ASCVD, despite new guidelines emphasizing their effect on reduced mortality. Statin therapy and aspirin prescriptions have shown only modest increases in recent years; however, gender and racial differences persist. There is a critical need to increase quality improvement and implementation programs in the primary care setting to address these gaps for secondary prevention of ASCVD.^25^

## Notes

Supplement. **eTable 1.** *ICD-9* and *ICD-10* Codes for ASCVD **eTable 2.** Characteristics Stratified by Study Year in Secondary Prevention Interventions Among ASCVD Patients Aged ≥21, 2006-2016
