# Supplementary material for: Trends in Provision of Medications and Lifestyle Counseling in Ambulatory Settings by Gender and Race for Patients With Atherosclerotic Cardiovascular Disease, 2006-2016
Source: JAMA Netw Open. 2023 Jan 19;6(1):e2251156. doi: 10.1001/jamanetworkopen.2022.51156 (PMC9857274; doi:10.1001/jamanetworkopen.2022.51156)
Supplement: Supplement. — eTable 1. ICD-9 and ICD-10 Codes for ASCVD eTable 2. Characteristics Stratified by Study Year in Secondary Prevention Interventions Among ASCVD Patients Aged ≥21, 2006-2016 [file jamanetwopen-e2251156-s001.pdf]

Supplemental Online Content

Mufarreh A, Shah AJ, Vaccarino V, Kulshreshtha A. Trends in provision of medications and lifestyle counseling in ambulatory settings by gender and race for patients with atherosclerotic cardiovascular disease, 2006-2016. *JAMA Netw Open*. 2023;6(1):e2251156. doi:10.1001/jamanetworkopen.2022.51156

eTable 1. *ICD-9* and *ICD-10* Codes for ASCVD

eTable 2. Characteristics Stratified by Study Year in Secondary Prevention Interventions Among ASCVD Patients Aged ≥21, 2006-2016

This supplemental material has been provided by the authors to give readers additional information about their work.

© 2023 Mufarreh A et al. *JAMA Network Open.*

eTable 1. *ICD-9* and *ICD-10* Codes for ASCVD

	**Code**	
**Name**	**ICD-10 (10/01/2015)**	**ICD-9**
Atherosclerosis	I70	440
Atherosclerosis of aorta	I70.1	440.1
Atherosclerosis of native arteries of the extremities	I70.2	440.2(0,1,2,3,4,9)
Atherosclerosis of unspecified type of bypass graft(s) of the extremities	I70.3	440.3
Atherosclerosis of autologous vein bypass graft(s) of the extremities	I70.4	440.31
Atherosclerosis of Nonautologous biological bypass graft(s) of the extremities	I70.5	440.32
Atherosclerosis of nonbiological bypass graft(s) of the extremities	I70.6	
Atherosclerosis of other type of bypass graft(s) of the extremities	I70.7	
Atherosclerosis of other arteries	I70.8	440.8
Other and unspecified atherosclerosis	I70.9	440.9
Angina pectoris	I20	413
Unstable angina	I20.0	411.1
Angina pectoris with documented spasm	I20.1	413.1

© 2023 Mufarreh A et al. *JAMA Network Open.*

Other forms of angina pectoris	I20.8	413.0
Angina pectoris, unspecified	I20.9	413.9
Acute myocardial infarction	I21	
ST elevation (STEMI) myocardial infarction of anterior wall	I21.0	410.(0,1,2)<br>410.1(0,1,2)
ST elevation (STEMI) myocardial infarction of inferior wall	I21.1	410.2(0,1,2)<br>410.3(0,1,2)<br>410.4(0,1,2)
ST elevation (STEMI) myocardial infarction of other sites	I21.2	410.5(0,1,2)<br>410.6(0,1,2)<br>410.8(0,1,2)
ST elevation (STEMI) myocardial infarction of unspecified site	I21.3	
Non-ST elevation (NSTEMI) myocardial infarction	I21.4	410.7(0,1,2)
Acute myocardial infarction, unspecified	I21.9	410.9(1,2,3)
Other type of myocardial infarction	I21.A	
Other acute ischemic heart disease	I24	
Other forms of acute ischemic heart disease	I24.8	411.89
Acute ischemic heart disease, unspecified	I24.9	
Family history of ischemic heart disease and other diseases of the circulatory system	Z82.4	V17.3

© 2023 Mufarreh A et al. *JAMA Network Open.*

Transient cerebral ischemic attacks and related syndromes	G45	
Vertebro-basilar artery syndrome	G45.0	435<br>435.(0,1,2,3,8,9)
Carotid artery syndrome (hemispheric)	G45.1	
Multiple and bilateral precerebral artery syndromes	G45.2	
Amaurosis fugax	G45.3	
Transient global amnesia	G45.4	
Other transient cerebral ischemic attacks and related syndromes	G45.8	
Transient cerebral ischemic attack, unspecified	G45.9	
Family history of stroke	Z82.3	V17.1
Nontraumatic subarachnoid hemorrhage (0)	I60<br>I60.(0,1,2,3,4,5,6,7,8,9)	430
Nontraumatic intracerebral hemorrhage (0)	I61.(0,1,2,3,4,5,6,7,8,9)	431
Other and unspecified nontraumatic intracranial hemorrhage	I62<br>I62.(0,1,9)	432.(0,1,9)
Cerebral infarction	I63<br>I63.(0,1,2,3,4,5,6,8,9)	

© 2023 Mufarreh A et al. *JAMA Network Open.*

Occlusion and stenosis of precerebral arteries, not resulting of cerebral infarction	I65<br>I65.(0,1,2,8,9)	433<br>433.0(0,1)<br>433.1(0,1)<br>433.2(0,1)<br>433.3(0,1)<br>433.8(0,1)<br>433.9(0,1)
Occlusion and stenosis of cerebral arteries, not resulting in cerebral infarction	I66<br>I66.(0,1,2,3,8,9)	434<br>434.0(0.1)<br>434.1(0,1)<br>434.9(0,1)
Cerebral atherosclerosis	I67.2	
Other peripheral vascular diseases	I73<br>I73.(0,1,8,9)	443<br>443.0<br>443.1<br>443.2(1,2,3,4,9)<br>443.8(1,2,9)<br>443.9

© 2023 Mufarreh A et al. *JAMA Network Open.*

eTable 2. Descriptive Characteristics Stratified by Study Year in Secondary Prevention Interventions Among ASCVD Patients Aged ≥21, 2006-2016

	N (%), millions										
	**2006**	**2007**	**2008**	**2009**	**2010**	**2011**	**2012**	**2013**	**2014**	**2015**	**2016**
**Total (weighted), N**	20.6	24.3	27.6	27.1	20.6	20.4	19.2	21.5	31.1	30.8	32.0
**Gender**											
**Men**	12.3 (59.8)	14.6 (60.2)	16.7 (60.5)	15.3 (56.6)	12.4 (60.1)	11.7 (57.2)	11.2 (58.3)	12.7 (59.0)	19.0 (61.1)	19.1 (62.0)	18.1 (56.6)
**Women**	8.3 (40.2)	9.7 (39.8)	10.9 (39.5)	11.7 (43.4)	8.2 (39.9)	8.7 (42.9)	8.0 (41.7)	8.8 (41.0)	12.1 (38.9)	11.7 (38.0)	13.9 (43.4)
**Race/Ethnicity**											
**NH White**	16.7 (94.3)	13.1 (91.5)	17.8 (91.5)	17.4 (92.5)	12.8 (85.9)	12.3 (89.8)	11.0 (91.8)	13.1 (88.5)	18.9 (91.7)	19.3 (84.4)	20.4 (89.7)
**NH Black**	1.0 (5.7)	1.2 (8.5)	1.6 (8.5)	1.4 (7.5)	2.1 (14.1)	1.4 (10.2)	1.0 (8.2)	1.7 (11.5)	1.7 (8.3)	3.6 (15.6)	2.3 (10.3)
**Hispanic**	1.9 (9.0)	3.0 (12.5)	1.9 (6.8)	2.5 (9.2)	1.3 (6.3)	2.6 (12.8)	1.5 (7.6)	0.9 (4.2)	3.6 (11.5)	2.3 (7.3)	4.0 (12.5)
**Age**											
**21-39**	0.2 (0.9)	0.2 (0.9)	0.6 (2.1)	0.3 (1.3)	0.4 (2.0)	0.4 (1.9)	0.2 (0.9)	0.3 (1.2)	0.4 (1.3)	0.9 (2.8)	0.9 (2.8)
**40-49**	1.3 (6.4)	1.3 (5.4)	1.6 (5.7)	1.5 (5.7)	0.9 (4.5)	1.4 (6.8)	0.8 (4.1)	0.8 (3.9)	1.0 (3.2)	0.8 (2.7)	1.7 (5.2)

© 2023 Mufarreh A et al. *JAMA Network Open.*

**50-59**	2.9 (14.1)	3.6 (14.9)	4.4 (15.9)	4.3 (15.7)	2.7 (12.9)	2.4 (11.9)	2.5 (13.2)	2.7 (12.6)	4.0 (12.9)	3.7 (11.9)	4.0 (12.4)
**60-75**	7.9 (38.2)	10.4 (42.8)	10.8 (39.1)	10.2 (37.7)	8.3 (40.3)	8.8 (42.9)	8.3 (43.2)	9.4 (44.0)	14.3 (45.9)	12.5 (40.5)	15.5 (48.5)
**≥75**	8.3 (40.3)	8.8 (36.0)	10.3 (37.2)	10.7 (39.6)	8.3 (40.3)	7.5 (36.5)	7.4 (38.6)	8.2 (38.3)	11.4 (36.8)	13.0 (42.3)	10.0 (31.1)
**Specialty**											
**Family**	5.6 (27.0)	3.8 (15.6)	3.8 (13.6)	3.6 (13.3)	2.9 (14.2)	4.1 (20.6)	3.3 (17.3)	4.3 (19.9)	5.5 (17.6)	6.1 (19.7)	7.3 (22.8)
**Internal**	4.1 (19.9)	6.0 (24.5)	5.7 (20.6)	5.4 (20.1)	5.3 (26.0)	4.4 (21.9)	4.7 (24.7)	3.8 (17.5)	6.0 (19.4)	7.3 (23.5)	3.0 (9.5)
**Cardiology**	8.3 (40.2)	9.2 (37.7)	14.4 (52.3)	11.7 (43.4)	8.7 (42.8)	7.3 (36.3)	7.2 (37.4)	8.5 (39.4)	14.0 (45.1)	17.5 (56.8)	11.2 (34.9)
**Census region**											
**Northeast**	5.2 (25.3)	4.1 (16.9)	7.3 (26.6)	4.1 (15.1)	3.1 (15.1)	5.1 (25.1)	4.2 (22.0)	4.9 (22.6)	7.4 (23.6)	7.3 (23.7)	5.6 (17.5)
**Midwest**	4.2 (20.5)	5.7 (23.3)	5.2 (18.7)	7.1 (26.1)	4.8 (23.4)	3.4 (16.5)	3.3 (17.4)	4.7 (21.8)	7.1 (22.8)	5.8 (18.7)	7.5 (23.5)
**South**	7.4 (35.6)	11.2 (46.1)	10.8 (39.1)	11.9 (43.9)	9.4 (45.5)	7.2 (35.2)	8.3 (43.4)	8.2 (38.0)	11.9 (38.2)	11.5 (37.1)	12.6 (39.3)
**West**	3.8 (18.6)	3.3 (13.7)	4.3 (15.6)	4.0 (14.9)	3.3 (15.9)	4.7 (23.2)	3.3 (17.2)	3.8 (17.6)	4.8 (15.4)	6.3 (20.4)	6.3 (19.7)
**Insurance**											

© 2023 Mufarreh A et al. *JAMA Network Open.*

**Private**	6.1 (31.0)	7.4 (31.2)	9.3 (34.4)	8.5 (32.5)	5.7 (28.4)	6.0 (30.0)	5.1 (28.8)	5.8 (28.2)	9.4 (31.7)	7.0 (24.0)	8.3 (28.4)
**Medicare**	11.1 (56.2)	13.6 (57.7)	16.4 (60.8)	16.5 (62.9)	13.3 (65.7)	12.9 (65.0)	11.6 (65.1)	12.9 (63.0)	18.5 (62.5)	19.7 (67.3)	18.9 (64.3)
**Medicaid/CHIP**	1.7 (8.4)	1.8 (7.6)	0.6 (2.3)	0.6 (2.1)	0.8 (4.1)	0.7 (3.3)	0.6 (3.3)	1.0 (5.0)	1.1 (3.8)	2.1 (7.2)	1.9 (6.3)
**Obesity**[1]	2.0 (9.7)	1.7 (7.0)	3.1 (11.2)	2.5 (9.2)	1.8 (8.9)	2.9 (14.3)	2.0 (10.5)	2.3 (10.7)	4.1 (13.2)	3.0 (9.8)	4.0 (12.4)
**Hypertension**	13.5 (65.5)	14.9 (61.3)	18.1 (65.7)	17.3 (63.9)	14.2 (68.8)	15.6 (76.5)	13.0 (68.0)	15.3 (71.2)	23.6 (75.9)	21.6 (70.0)	24.0 (74.9)
**Diabetes**	6.4 (30.8)	5.6 (23.1)	6.5 (23.6)	7.2 (26.6)	5.2 (25.0)	6.0 (29.6)	5.3 (27.9)	6.3 (29.1)	8.9 (28.5)	9.9 (32.0)	12.6 (39.4)
**Hyperlipidemia**	11.4 (55.2)	12.1 (49.7)	14.8 (53.6)	15.5 (57.1)	11.5 (55.9)	11.1 (54.5)	10.3 (53.5)	12.0 (55.9)	20.3 (65.1)	18.7 (60.6)	20.7 (64.5)
**Cerebrovascular disease**	3.8 (18.4)	5.2 (21.4)	5.7 (20.6)	6.5 (24.0)	4.1 (19.8)	5.1 (25.2)	5.0 (26.0)	5.4 (25.1)	6.8 (21.7)	5.9 (19.0)	7.0 (21.7)
**Tobacco use**[2]	2.6 (13.1)	3.2 (13.8)	2.5 (9.7)	3.2 (12.6)	2.5 (12.2)	2.6 (13.8)	2.4 (13.0)	3.0 (14.1)	4.2 (13.9)	3.3 (10.9)	4.4 (13.9)
**Tobacco education**	1.2 (5.9)	1.1 (3.7)	1.0 (3.7)	1.6 (6.0)	1.2 (5.8)	1.0 (4.8)	0.7 (3.6)	1.4 (6.3)	1.4 (4.5)	1.7 (5.6)	2.1 (6.7)
**Renal failure**	0.6 (2.8)	0.7 (2.9)	1.3 (4.7)	1.0 (3.8)	1.2 (5.6)	2.1 (10.4)	0.8 (4.1)	1.1 (5.1)	2.2 (7.1)	4.9 (15.8)	2.4 (7.5)
**Aspirin**	8.5 (41.3)	9.5 (39.1)	12.4 (45.1)	12.0 (44.4)	9.1 (43.9)	7.7 (37.7)	7.4 (38.8)	10.0 (46.4)	16.0 (51.5)	15.9 (51.5)	15.2 (47.5)

© 2023 Mufarreh A et al. *JAMA Network Open.*

**Statin**	9.3 (45.3)	9.2 (37.8)	12.8 (46.6)	12.9 (47.6)	10.8 (52.3)	9.1 (44.6)	8.8 (45.8)	10.7 (49.8)	18.8 (60.3)	17.2 (55.7)	14.9 (46.5)
**Lifestyle Counseling**	6.8 (33.0)	5.3 (21.9)	8.2 (29.7)	6.3 (23.4)	4.5 (21.9)	4.8 (23.7)	2.7 (13.8)	4.0 (18.6)	5.2 (16.6)	8.2 (26.7)	7.1 (22.3)

Values presented as weight frequency number (N) and percentage (%)
Abbreviations: ASCVD, atherosclerotic cardiovascular disease; NH, non-Hispanic; ACC/AHA, American College of Cardiology and American Heart Associations
[1]Body mass index (kg/m^2) ≥ 30
[2]Smoking cigarettes/cigars, using snuff, or chewing tobacco

© 2023 Mufarreh A et al. *JAMA Network Open.*
